# PGS-Depot: a comprehensive resource for polygenic scores constructed by summary statistics based methods

**DOI:** 10.1093/nar/gkad1029

**Published:** 2023-11-11

**Authors:** Chen Cao, Shuting Zhang, Jianhua Wang, Min Tian, Xiaolong Ji, Dandan Huang, Sheng Yang, Ning Gu

**Affiliations:** Key Laboratory for Bio-Electromagnetic Environment and Advanced Medical Theranostics, School of Biomedical Engineering and Informatics, Nanjing Medical University, Nanjing, Jiangsu 211166, China; Key Laboratory for Bio-Electromagnetic Environment and Advanced Medical Theranostics, School of Biomedical Engineering and Informatics, Nanjing Medical University, Nanjing, Jiangsu 211166, China; Department of Pharmacology, School of Basic Medical Sciences, Tianjin Medical University, Tianjin 300203, China; Key Laboratory for Bio-Electromagnetic Environment and Advanced Medical Theranostics, School of Biomedical Engineering and Informatics, Nanjing Medical University, Nanjing, Jiangsu 211166, China; Department of Biostatistics, Centre for Global Health, School of Public Health, Nanjing Medical University, Nanjing, Jiangsu 211166, China; Department of Pharmacology, School of Basic Medical Sciences, Tianjin Medical University, Tianjin 300203, China; Department of Biostatistics, Centre for Global Health, School of Public Health, Nanjing Medical University, Nanjing, Jiangsu 211166, China; Key Laboratory for Bio-Electromagnetic Environment and Advanced Medical Theranostics, School of Biomedical Engineering and Informatics, Nanjing Medical University, Nanjing, Jiangsu 211166, China; Medical School, Nanjing University, Nanjing, Jiangsu 210093, China

## Abstract

Polygenic score (PGS) is an important tool for the genetic prediction of complex traits. However, there are currently no resources providing comprehensive PGSs computed from published summary statistics, and it is difficult to implement and run different PGS methods due to the complexity of their pipelines and parameter settings. To address these issues, we introduce a new resource called PGS-Depot containing the most comprehensive set of publicly available disease-related GWAS summary statistics. PGS-Depot includes 5585 high quality summary statistics (1933 quantitative and 3652 binary trait statistics) curated from 1564 traits in European and East Asian populations. A standardized best-practice pipeline is used to implement 11 summary statistics-based PGS methods, each with different model assumptions and estimation procedures. The prediction performance of each method can be compared for both in- and cross-ancestry populations, and users can also submit their own summary statistics to obtain custom PGS with the available methods. Other features include searching for PGSs by trait name, publication, cohort information, population, or the MeSH ontology tree and searching for trait descriptions with the experimental factor ontology (EFO). All scores, SNP effect sizes and summary statistics can be downloaded via FTP. PGS-Depot is freely available at http://www.pgsdepot.net.

## Graphical Abstract

**Graphical Abstract:**
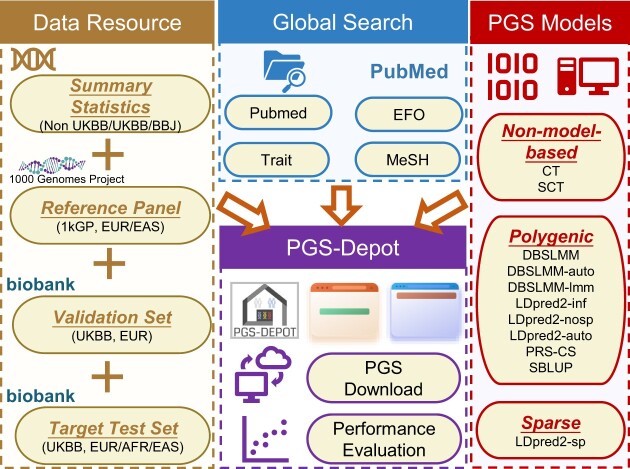

## Introduction

Complex traits, such as cardiovascular disease (1), psychiatric disorders (2), and different types of cancers (3) do not follow simple Mendelian inheritance laws. Accurate genetic prediction of complex disease may facilitate population-scale risk stratification and improve early-stage diagnosis and intervention (4–6). Polygenic score (PGS), which is a weighted summation of the estimated effect sizes of single nucleotide polymorphisms (SNPs) across the whole genome, is a common measurement for the prediction of complex traits from genotype data (7). PGS is commonly called polygenic risk score (PRS) or genetic risk score (GRS) when the trait of interest is a binary trait of disease status. After proposed by Purcell *et al.*, PGS has been extensively employed for disease risk stratification and precision clinical decision (8). Currently, most PGSs are constructed using European populations (EUR), but their transferability to other population groups, such as East Asian (EAS), South Asian, or African (AFR), is also an important application which is limited by reduced accuracy (9,10). This loss of prediction performance when EUR models are directly applied to different ancestries might be caused by genetic differences such as linkage disequilibrium (LD), allele frequencies, and/or variations in genetic architecture between populations (11,12). Biobank-scale data such as UK Biobank (UKBB), Biobank of Japan (BBJ) and China Kadoorie Biobank (CKB), include a large number of individuals (i.e. sample size larger than 500 000), which improves the power of downstream genome-wide association studies (GWAS) and therefore the prediction performance of PGS (13–16). The recent availability of biobanks focusing on non-European individuals has paved the way for the evaluation of PGS prediction accuracy on both in-ancestry and cross-ancestry populations (17).

The prediction performance of PGS depends strongly on the assumptions made regarding the distribution of SNP effect sizes (18). In addition, GWAS summary statistics (i.e. variant, effect size, standard error and *P*-value for a specific trait) are more commonly available than individual-level data (19,20). Given these two points, many PGS methods utilizing summary statistics have been proposed, which assume different distributions of SNP effect sizes (i.e. polygenic or sparse) and/or different statistical inference strategies (i.e. frequentist or Bayesian). Polygenic methods assume that all SNPs have non-zero effects and follow a specific distribution such as the normal distribution (21–24). Meanwhile, sparse methods assume that only a small proportion of SNPs have non-zero effects (21). As different traits have different genetic architectures, no single modeling assumption is suitable for all traits. This motivates the need for a comprehensive database for cataloging and evaluating the scores, prediction performance and transferability of multiple PGS methods.

To date, there exist multiple open-access databases for GWAS summary statistics, including the NHGRI-EBI GWAS Catalog (25), OpenGWAS (26) and GWAS Central (27). On the other hand, the PGS Catalog, which includes 619 traits for different goals compared to PGS-Depot, is the only PGS database that indexes published PGS along with metadata describing how the scores were developed and evaluated, with data derived from curation or author submissions (28). The limited number of traits and PGS methods publicly available may hinder the utility of PGS and GWAS to epidemiologists, physicians and geneticists. To address these limitations, we propose PGS-Depot as a comprehensive resource that implements 11 summary statistics-based PGS methods for 1564 complex traits. The performance of each method is evaluated using Pearson *R*^2^ or area under curve (AUC), and the prediction performance for both in-ancestry and cross-ancestry populations is estimated. PGS-Depot is an open access resource which is freely available at http://www.pgsdepot.net.

## Materials and methods

### Summary statistics curation

**Figure 1.**
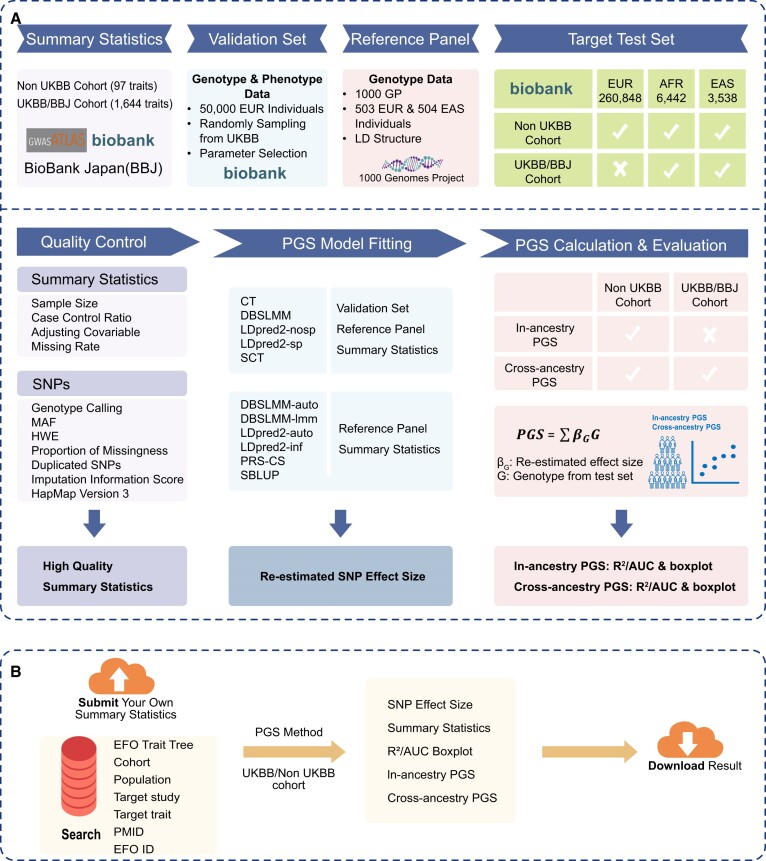
An overview of PGS-Depot. **(A)** The pipeline of PGS-Depot, including data source, data processing and output. First, we performed quality control for the collected summary statistics. We kept only 5585 summary statistics consisting of both quantitative and binary traits. Second, we fitted models for 11 PGS methods. 50 000 UKBB individuals were randomly sampled as a validation set, and the 1000 Genomes Project was used as a reference panel. Finally, we calculated PGSs and evaluated their performance. The test set consisted of three ancestry groups from UKBB. **(B)** A workflow of PGS-Depot. Users can query traits in seven ways, including by experimental factor ontology (EFO) trait tree, cohort, population, target study, target trait name, PMID and EFO ID. Users can also upload their own summary statistics for analysis. Users can choose PGS methods based on their in- and/or cross-ancestry prediction performance and download results by method and population.

PGS-Depot contains 5585 high quality summary statistics divided between 1933 statistics for quantitative traits and 3652 for binary traits, which have been curated from 1564 traits in both EUR and EAS populations (Figure 1). To guarantee the availability, validity and accuracy of PGS in the database, we retained those summary statistics: (i) with a sample size larger than 2000; (ii) whose traits are recorded in UKBB; (iii) whose covariates and stratification variables are also measured in UKBB and (iv) with a missing rate of less than 50% in UKBB. For binary traits, we additionally restricted traits to those with a case-control ratio higher than 1:500. We divided these summary statistics into three categories according to their data source: (i) non-UKBB EUR cohort; (ii) UKBB cohort and (iii) BBJ cohort. For summary statistics that do not include a minor allele frequency (MAF), we directly used the MAF of the corresponding ancestry group in the 1000 Genomes Project (1000GP).

### PGS methods

PGS models vary based on their model assumptions. The prediction efficacy of different PGS models depends on the congruence between the model's effect size assumptions and the trait's genetic architecture. Based on different distribution assumption of effect size, we classified the methods into three categories: non-model-based, polygenic and sparse methods. Therefore, PGS-Depot encompasses scores constructed using 11 methods with different model assumptions detailed in Supplementary Table S1. Non-model-based methods such as CT and SCT employ independent SNPs with large effect size without making prior assumptions about the effect sizes. We divided polygenic methods into two subcategories: infinitesimal models like DBSLMM-lmm, LDpred2-inf and SBLUP (assuming a uniform variance across all SNP effect sizes conforming to a normal distribution), and others like DBSLMM-auto, PRS-CS, LDpred2-auto and LDpred2-nosp (where each effect size follows a normal distribution within its unique variance). Sparse methods like LDpred2-sp assume that only a small fraction of SNPs is associated with the trait.

Different model assumptions necessitate varying dataset requirements for each method. Specifically, some methods require only a reference panel, while others demand both a reference panel and a validation set. For our analysis, we used six different methods: DBSLMM-auto, DBSLMM-lmm, LDpred2-auto, LDpred2-inf, SBLUP and PRS-CS. These were fitted using summary statistics derived from a specific population alongside a reference panel corresponding to the same population. We used the genotypes from 1000GP as a reference panel, which contains 503 EUR and 504 EAS individuals. These six methods utilize block-diagonal LD matrices to reduce computational cost with three different strategies: (i) DBSLMM and PRS-CS construct a block-diagonal LD matrix following (29); (ii) SBLUP fixes the LD window size to 1Mb; and (iii) LDpred2 sets the LD window size to 3cM. For the remaining methods, we additionally used a validation set to select optimal parameter settings. Specifically, to reduce the computational burden, we used the reference panel to estimate SNP effect sizes and used the validation set to select the best parameters. The details of the parameter settings and inputs for the 11 methods are shown below.

For CT, we selected three hyper-parameters from among 2800 possible combinations, which are drawn from 50 *P*-value thresholds, four window sizes (50, 100, 200 and 500 kb), and seven *R*^2^ values (0.01, 0.05, 0.1, 0.2, 0.5, 0.8 and 0.9). SCT extends CT by fitting a penalized regression. To reduce computational costs, we fitted four LDpred2 models separately to each chromosome. LDpred2-inf uses a closed-form estimator to fit its LMM. For LDpred2-nosp, we tuned two hyper-parameters by considering 21 different *P*-values ranging from 10^−5^ to 1 on the log-scale, and three heritability values $\{ {0.7,1.0,1.4} \} \times h_{chr}^2$. LDpred2-sp fits the same model as LDpred2-nosp but constrains some SNP effects to zero. Without additional validation dataset, LDpred2-auto automatically infers the two hyper-parameters. For the methods above, we used the R package *bigsnpr* (v. 1.12.2) to fit models. DBSLMM classified SNPs by effect size into a group of large effect SNPs and a group of small effect SNPs. To choose the two hyperparameters in DBSLMM, we used a validation set to select between three *P*-value thresholds (10^−4^, 10^−5^, or 10^−6^) and three heritability values $\{ {0.8,1.0,1.2} \} \times {h^2}$. On the other hand, while DBSLMM adopts a distinct approach, DBSLMM-auto uses window size as 1 Mb, *R*^2^ as 0.2, and *P*-value as 10^−6^ to define the large effect SNP. Unlike DBSLMM, DBSLMM-lmm fits a linear mixed model (LMM). For the three DBSLMM methods, we used the *DBSLMM* software (v. 0.3) to estimate effect sizes and the LD Score Regression (LDSC) software (v. 1.0) to estimate SNP heritability (24,30). Using *GCTA* (v. 1.94.1), SBLUP fits an LMM with heritability estimated by LDSC. Although LDpred2-inf, DBSLMM-lmm, and SBLUP fitting the LMM, they used different strategies to construct LD matrix. PRS-CS places continuous shrinkage (CS) on the SNP effect size, for which we used 1000 sampling iterations with 500 burn-in iterations as a default.

### Quality control for UKBB cohort

For the quality control (QC) of UKBB EUR individuals, we retained individuals (i) whose genotypes were successfully measured, (ii) who are included in the genotype principal component (PC) computation and (iii) who have a white British ancestry for in-ancestry PGS or EAS/AFR ancestry for cross-ancestry PGS (2,16) (Figure 1). In addition, we excluded individuals (i) who have more than 10 putative third-degree relatives based on the kinship table, (ii) who have sex chromosome aneuploidy and (iii) who are redacted and thus do not have a corresponding ID in the phenotype data. For SNP QC, we retained SNPs with a genotype calling confidence larger than 0.9. We filtered out SNPs (i) with a MAF <0.01, (ii) with a Hardy–Weinberg equilibrium (HWE) test *P*-value <10^−7^, (iii) with an imputation information score <0.8, (iv) with a proportion of missingness (*P*_m_) >0.05 or (v) that are a duplicated SNP. Following (5,22), to ensure scalability for all PGS methods (i.e. PRS-CS), PGS-Depot only includes about one million SNPs in HapMap phase 3 (HM3) version (22,31).

### PGS for non-UKBB cohort

PGS-Depot includes 200 summary statistics estimated without UKBB, of which 75 summary statistics and 31 traits are quantitative traits, and 125 statistics and 66 traits are binary traits (Supplementary Table S2). For these statistics, we applied all 11 PGS methods to re-estimate the effect size. As different summary statistics were previously estimated with differing covariates and/or stratifications, we adjusted the covariates and/or stratified the data appropriately to match. Specifically, for each quantitative trait, we fitted a linear regression to remove the effects of the top 20 genotype PCs and their corresponding covariates. We transformed phenotype residuals to a standard normal distribution through quantile-quantile transformation. For each binary trait, we directly fitted a logistic regression model for the top 20 genotype PCs and their corresponding covariates to obtain their effect sizes. For binary traits, we treated self-reported or ICD10 cases as positive and the remaining as negative (2,16). When a summary statistic was estimated by stratification analysis, we fitted the model to the corresponding stratification in the validation set. For example, we used a sample of 18531 females as the validation set for body mass index (BMI) stratified by sex.

For Non-UKBB PGSs, we used a validation set to select the best parameter combination and a test set to evaluate the prediction performance of each of the 11 PGS methods. Both the validation and test sets were drawn from UKBB. We randomly selected 50 000 UKBB EUR individuals as a validation set. The test set consists of the remaining UKBB data with 270828 individuals, of which there are 260 848 EUR, 6442 AFR and 3538 EAS individuals (Supplementary Table S3). To evaluate in-ancestry performance, we performed 100 bootstrap samples consisting of 5000 EUR individuals each. To evaluate cross-ancestry performance, due to limited sample size we performed 100 bootstrap samples with 500 AFR or EAS individuals per sample. Note that the limited number of AFR and EUR samples caused four of our cross-ancestry tests for binary traits with stratified variables to fail. Specifically, due to the variation of sample size between the three ancestries, the prevalence in many binary traits was different. This imbalance could lead to higher cross-ancestry prediction performance in EAS or AFR. We evaluated the performance of all 11 methods using *R*^2^ for quantitative traits and AUC for binary traits and used boxplots to show the *R*^2^ or AUC of the 100 bootstrap iterations.

### PGS for UKBB cohort or BBJ cohort

The remaining summary statistics were estimated using the UKBB or BBJ cohort, comprising two categories: (i) statistics relying solely on the two biobank-scale datasets; (ii) statistics that employ the two biobank-scale datasets to perform meta-analysis. For statistics encompassing UKBB participants, the UKBB validation set was omitted to mitigate potential over-fitting. BBJ is the largest disease biobank, recruiting 260 000 patients representing 440 000 cases in 51 diseases (14). For the summary statistics estimated by BBJ participants, a distinction in disease prevalence was observed when compared to the EAS segment of the UKBB cohort. Furthermore, the limited EAS sample size in UKBB, totaling 3538, precluded a division into distinct validation and test sets. Given these constraints, our evaluation adopted six models: DBSLMM-auto, DBSLMM-lmm, LDpred2-auto, LDpred2-inf, PRS-CS and SBLUP, given that these models only required either a EUR or EAS reference panel.

### Implementation of PGS-Depot

The back end of PGS-Depot was developed in the Java-based Spring Boot web framework. The front-end was developed with the Vue.js framework, and the user interface was built with the Element framework for Vue.js. A MySQL database is used to rapidly retrieve curated GWAS summary statistics and SNP effect sizes for different PGS methods.

## Results

### Design and organization of PGS-Depot

**Figure 2.**
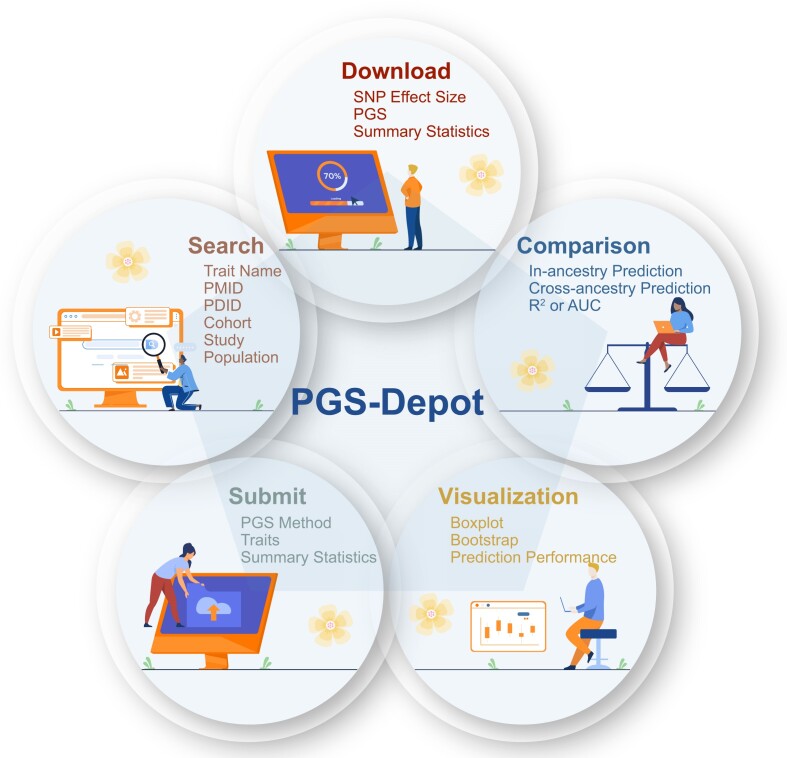
Summary of functional modules in PGS-Depot. PGS-Depot consists of five modules: search, download, comparison, visualization and submit.

PGS-Depot organizes and presents all results (i.e. summary statistics and scores from all PGS methods) under the webpages: *PGS*, *Non UKBB PGS*, *Traits*, *Publications*, *Search, Submit, About* and *Help* (Figure 2). On the *PGS* and *Non UKBB PGS* pages, we provide information for eleven different properties: GWAS summary statistics ID in PGS-Depot (PDID), reported trait, experimental factor ontology (EFO) ID (32), EFO ontology trait, sample size, number of controls, number of cases, population, cohort, PGS methods, and PMID. We specifically indicate the suitable methods for constructing each given PGS. The *Traits* page lists the PDID, reported trait, trait label, trait ontology ID, trait ID, population and sample size for each trait. We also provide a pie chart to show the proportion of traits among 26 trait categories. The *Publications* page presents the PMID, PMCID, title, first author, journal, year and DOI of each publication. Together, these pages organize PGS according to four different aspects, and not only provide searching and sorting functions but also allow the summary information for all PGSs to be downloaded. On the *Search* page, we provide four options for searching by trait, population, cohort and publication. On the *Submit* page, we provide an interface to upload the user's summary statistics for analysis. Users must provide their email address, trait, sample size and summary statistic information (i.e. SNP name, effect allele, non-effect allele, beta and *P*-value). Furthermore, we’ve incorporated a ‘private data switch’, allowing users to designate data confidentiality, with an adjacent description ensuring the secure handling and prompt deletion of any private data post-analysis. For restricted datasets, such as those from the Psychiatry Genomics Consortium (PGC) (33–35), we compute the PGS, dispatch results via email and erase the PGS and summary statistics from our system within seven days. The *About* page provides a detailed introduction to PGS-Depot, and the *Help* page provides a case study showing the results presented by PGS-Depot.

### Usage and interface of PGS-Depot

To find PGS results, users can search from different pages with different querying methods. Results are presented differently between the non-UKBB cohort and UKBB/BBJ cohort. For the non-UKBB cohort, we provide the following information: (i) summary statistic information such as the reported trait, EFO trait, population, sample size, PMID, number of cases, number of controls and link to corresponding publication; (ii) the EFO ID and EFO ontology; (iii) the top 100 significant SNPs; and (iv) three boxplots that compare the in- and cross-ancestry prediction performance of the 11 PGS models. For the UKBB/BBJ cohort, we provide the first three pieces of information. Users can download the SNP effect sizes estimated by the six PGS methods, scores for EUR, AFR and EAS populations generated by each method, and the corresponding summary statistics used to generate the scores. The SNP effect size file contains three necessary pieces of information: SNP ID, effective allele and effect size, which can be directly used with PLINK (36).

The search interface of PGS-Depot utilizes the Elasticsearch search engine to enable searching by PDID, trait name and PMID. Users can also search the contents of any column (such as publication year). Complete search results can be downloaded for further analysis using the download button.

### Case study: Alzheimer's disease neuropathologic change (PDID: PD05427)

**Figure 3.**
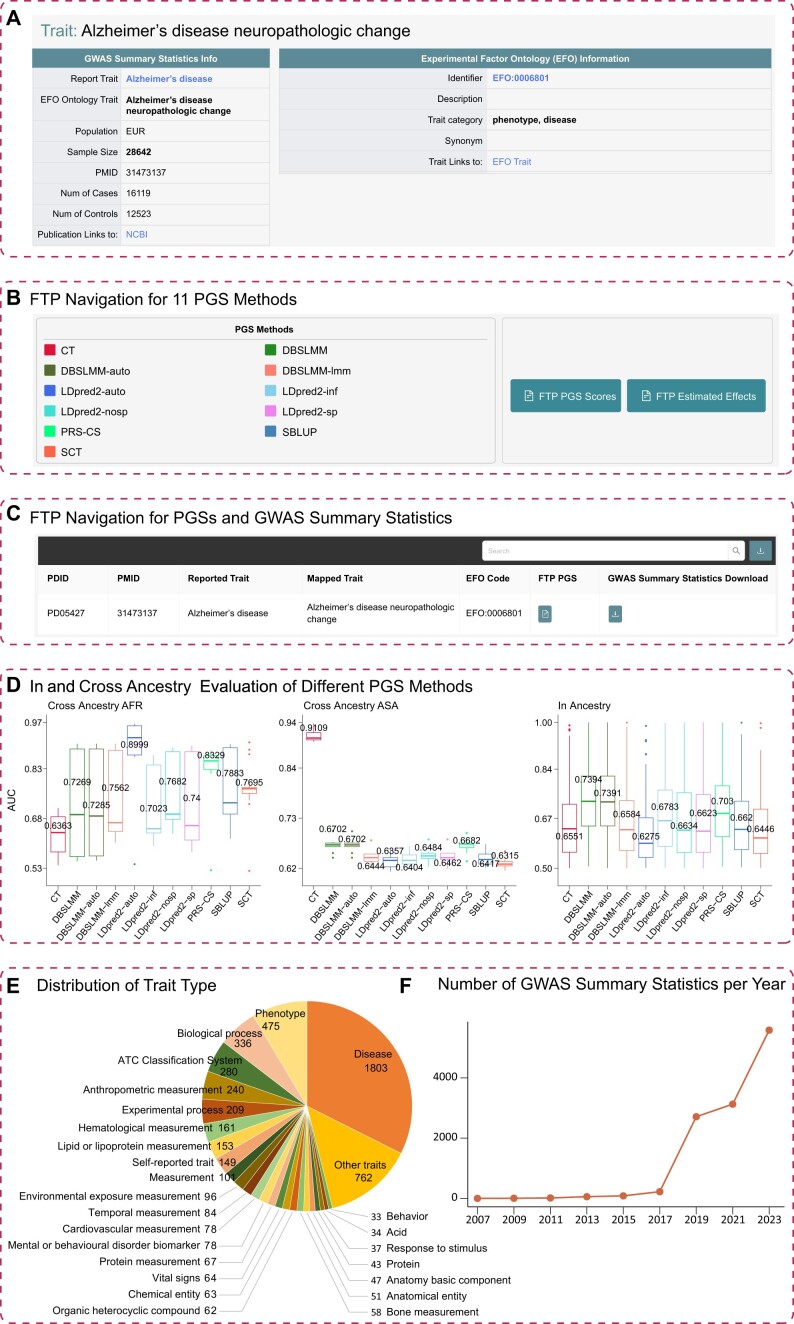
An example from PGS-Depot for Alzheimer's disease. **(A)** The information page for Alzheimer's disease, including summary information and EFO information. **(B)** FTP navigation for downloading PGSs by method. **(C)** FTP navigation for downloading PGSs and summary statistics by trait. **(D)** In and cross ancestry evaluation of different PGS methods. **(E)** Pie chart showing the proportion of traits from 26 trait categories. **(F)** Line graph showing the number of GWAS summary statistics published per year (2007–2023).

We treat Alzheimer's disease as an example to demonstrate the usability of the resource and the information provided for each summary statistic (Figure 3). First, on the *Non-UKBB Cohort* or *Search* page, users can use the PMID, trait name, or PDID to find the appropriate information page. On the information page, PGS-Depot provides summary information, EFO information and results for each of the 11 implemented PGS methods including prediction performance, FTP links for downloading effect sizes and a summary of the most significant SNPs. In particular, using summary statistics from 28 642 EUR individuals consisting of 16 119 cases and 12 523 controls (37), we re-estimated SNP effect sizes with each of the 11 PGS methods to provide prediction scores for three ancestry groups. This page also provides a link to directly download the relevant summary statistics. Boxplots are provided to visualize the prediction performance of the 11 PGS methods on each population. In this example, it is well known that the top SNP for Alzheimer's disease is the *cis*-SNP of the gene *APOE* located on chromosome 19 at position q13.32 (Supplementary Table S4).

### Comparison to PGS-Catalog

Compared to PGS-Catalog, which is the most popular PGS resource currently available, PGS-Depot is a more comprehensive database with multiple advantages. First, PGS-Catalog only includes 3688 scores for 619 traits, whereas PGS-Depot has collected 34 510 scores for 1564 traits. Second, unlike the PGS Catalog, where methods are curated or from author submissions, PGS-Depot applies 11 methods tailored to a specific trait. Third, PGS-Catalog stores scores estimated from the same summary statistics using different methods on different pages, which makes it difficult to compare methods. PGS-Depot instead displays scores from different methods on the same page, which makes downloading scores more user-friendly and makes selecting the best method easier. Finally, PGS-Depot provides boxplots to visualize the performance of different methods on both in- and cross-ancestry populations.

## Discussion

With the rapid growth of GWAS summary statistics and the increasing usage of PGS in genetic epidemiology, it is difficult for physicians to choose the best method (i.e. with regards to accuracy or transferability) for a given use case. To meet this need, we have developed PGS-Depot as a comprehensive database for evaluating the prediction accuracy and transferability of PGS methods. PGS-Depot features 11 different methods based on both polygenic and sparse assumptions which are suited to different genetic architectures, and provides scores for 1564 complex traits, including both quantitative and binary traits. For usability, PGS-Depot provides reports and visualizations for SNP effect sizes, a global search engine, sorting of tables by column, and FTP links for users to easily download PGS data.

Although PGS-Depot has many advantages, some limitations and future directions should be addressed. First, we’ve included summary statistics from studies with smaller sample sizes, particularly those with fewer than 4000 samples. Although the sample size of these studies is not very large, their PGS can be used in subsequent studies, especially those with non-overlapping samples. Second, several recent methods have been developed for computing cross-ancestry PGS utilizing different strategies such as a linear combination of PGSs from different ancestries and/or different methods (i.e. PRS-CSx, PolyPred and PGSagg) (5,31,38); transferring learning to fine-tune existing models on new ancestries (10); and re-estimation of effect sizes based on cross-ancestry genetic correlation (39). Finally, with the fast-growing need for cross-ancestry predictions and the emergence of non-EUR GWAS datasets, we will update PGS-Depot bimonthly by integrating more cross-ancestry PGS methods and more GWAS summary statistics in the future. In addition, users will be able to use these new methods when estimating PGSs on their own uploaded summary statistics.

We employed the HM3 version's SNPs, totalling about 1 million, to apply the 11 methods across all summary statistics. Despite using a sparser SNP setting, the predictive performance appears to be comparable between dense and sparse configurations. First, for most complex traits, common variants contribute a larger fraction of phenotypic variance as compared to rare variants (40). Second, accurate effect size estimation is crucial to phenotype prediction. The effect sizes of common variants can be estimated much more accurately than in the case of rare variants, which results in higher statistical power (22). In addition, including SNPs with low MAF would substantially increase the number of SNPs included in the PGS models, leading to an extremely heavy computational burden for some PGS methods. For example, PRS-CS and LDpred2 computation becomes notably prolonged when analyzing more than one million SNPs (5,27). In our prior investigation of six PGS methods across 25 binary traits, the performance improvement using a dense SNP set averages around 0.00%, with a range from –0.06% to 0.07% (5).

In summary, PGS-Depot is a comprehensive PGS resource that includes methods with non-model-based, sparse and polygenic assumptions, evaluates prediction performances on different ancestries, and provides SNP effect sizes in a format directly usable in PLINK. We believe that PGS-Depot will greatly expand the understanding of the genetic architecture underlying cancer survival and other complex diseases for geneticists and physicians worldwide, further providing an important resource for precision medicine.

## Supplementary Material

gkad1029_Supplemental_File

## Data Availability

Code for UKBB data processing is on Github (https://github.com/biostat0903/PGS-Depot) and FigShare (https://doi.org/10.6084/m9.figshare.24406210.v1). All relevant data is available through the PGS-Depot website (http://www.pgsdepot.net/). No new data were generated or analysed in support of this research.
