# Correction to ‘PGS-Depot: a comprehensive resource for polygenic scores constructed by summary statistics based methods’

**DOI:** 10.1093/nar/gkae351

**Published:** 2024-04-25

This is a correction to: Chen Cao, Shuting Zhang, Jianhua Wang, Min Tian, Xiaolong Ji, Dandan Huang, Sheng Yang, Ning Gu, PGS-Depot: a comprehensive resource for polygenic scores constructed by summary statistics based methods, *Nucleic Acids Research*, Volume 52, Issue D1, 5 January 2024, Pages D963–D971, https://doi.org/10.1093/nar/gkad1029

The statement ‘On the other hand, PGS Catalog is the only PGS database currently available which only supports the four methods of clumping and threshold (CT), LDpred, PRSice2, and metaGRS for 619 traits’ is inaccurate and should be corrected to ‘On the other hand, the PGS Catalog, which includes 619 traits for different goals compared to PGS-Depot, is the only PGS database that indexes published PGS along with metadata describing how the scores were developed and evaluated, with data derived from curation or author submissions.’

The statement ‘Second, PGS Catalog only includes four PGS methods, whereas PGS-Depot includes 11 methods’ is also inaccurate and should be corrected to ‘Second, unlike the PGS Catalog, where methods are curated or from author submissions, PGS-Depot applies 11 methods tailored to a specific trait’.

These corrections do not affect the results, discussion and conclusions presented in the article. The published article has been updated.

